# Optimal management strategy of insecticide resistance under various insect life histories: Heterogeneous timing of selection and interpatch dispersal

**DOI:** 10.1111/eva.12550

**Published:** 2017-11-02

**Authors:** Masaaki Sudo, Daisuke Takahashi, David A. Andow, Yoshito Suzuki, Takehiko Yamanaka

**Affiliations:** ^1^ Statistical Modeling Unit Institute for Agro‐Environmental Sciences NARO, Tsukuba Ibaraki Japan; ^2^ Tea Pest Management Unit Institute of Fruit Tree and Tea Science NARO, Kanaya, Shimada, Shizuoka Japan; ^3^ Department of Mathematics and Mathematical Statistics Umeå University Umeå Sweden; ^4^ Department of Entomology University of Minnesota St. Paul MN USA; ^5^ Graduate School of Life and Environmental Sciences Kyoto Prefectural University, Shimogamo Kyoto Japan

**Keywords:** high‐dose/refuge, interpatch dispersal, pesticide rotation, population‐based model, pyramiding, selection pressure

## Abstract

Although theoretical studies have shown that the mixture strategy, which uses multiple toxins simultaneously, can effectively delay the evolution of insecticide resistance, whether it is the optimal management strategy under different insect life histories and insecticide types remains unknown. To test the robustness of this management strategy over different life histories, we developed a series of simulation models that cover almost all the diploid insect types and have the same basic structure describing pest population dynamics and resistance evolution with discrete time steps. For each of two insecticidal toxins, independent one‐locus two‐allele autosomal inheritance of resistance was assumed. The simulations demonstrated the optimality of the mixture strategy either when insecticide efficacy was incomplete or when some part of the population disperses between patches before mating. The rotation strategy, which uses one insecticide on one pest generation and a different one on the next, did not differ from sequential usage in the time to resistance, except when dominance was low. It was the optimal strategy when insecticide efficacy was high and premating selection and dispersal occur.

## 1 INTRODUCTION

Insecticides have a long history in the struggle against resistance evolution. For more than 100 years, people have recognized how easily insects can acquire resistance if the insecticide continuously kills the majority of the target population, thereby selecting resistant individuals (Melander, 1914). The situation became far more serious with the widespread use of synthetic organic chemical insecticides such as DDT, which rapidly broke down due to resistance in many important pests, including housefly (*Musca domestica*, Muscidae), malarial mosquitoes (*Anopheles gambiae*, Culicidae), and body lice (*Pediculus corporis*, Pediculidae; Lindquist & Wilson, 1948; Metcalf, 1983; Wilson & Gahan, 1948). Since then, theoretical and empirical ecologists have developed several strategies to manage resistance evolution for the many insecticides released over the decades having different active ingredients.

The first influential strategy was to apply two or more insecticides alternating in turn, that is, rotation (Coyne, 1951). The basic idea of rotation is simple: Relax the selection pressure of a single toxin by applying a different toxin for some period of time. Several field and laboratory experiments have confirmed the effectiveness of rotation (Attique, Khaliq, & Sayyed, 2006; Georghiou, 1983; Immaraju, Morse, & Hobza, 1990), and it still plays an important role in resistance management (Bielza et al., 2008; Cloyd, 2010).

Another strategy, the high‐dose/refuge (HDR) strategy, has been playing a significant role in genetically engineered *Bt* crop resistance management. It does not require multiple toxins, but does require a spatial arrangement for application. Specifically, some portion of the habitat must not receive the toxin, and this “refuge” reduces the selection pressure on the population (Ives & Andow, 2002). Scientist strongly recommended the HDR strategy to governmental organizations prior to the first commercial use of a *Bt* crop (Alstad & Andow, 1995; Gould, 1998). The refuge should be near the control area, which is treated with a high‐dose insecticide (e.g., several *Bt* crops). It is assumed that the high‐dose insecticide can kill susceptible homozygotes (*SS*) and *RS* heterozygotes, leaving only an extremely small number of resistant homozygotes (*RR*) in the treated area. These *RR* homozygotes will mate with the excess amount of susceptible homozygotes immigrating from the refuges and will produce mostly heterozygous offspring, which would be eliminated by the high‐dose toxin (Alstad & Andow, 1995; Roush & McKenzie, 1987). So far, the HDR strategy has successfully prevented rapid resistance evolution more than 15 years if the refuges properly used (Huang, Andow, & Buschman, 2011; Tabashnik, Brévault, & Carrière, 2013).

Mixture of toxins is another important strategy both for conventional insecticide applications and for *Bt* crop management (known as *Bt* toxin pyramiding). Although a mixture of multiple toxins has conventionally been used to enhance pest control, empirical and theoretical work suggests that it is also effective to delay or prevent resistance evolution if cross‐resistance is negligible (Comins, 1986; Curtis, 1985; Pimentel & Bellotti, 1976). In theory, it shares a similar mechanism as the HDR strategy (Ives, Glaum, Ziebarth, & Andow, 2011). If the multiple toxins have no cross‐resistance, the survivors must be doubly resistant and will be extremely small in number. They will mate with large numbers of the immigrant, unselected population from outside the field.

In addition to these three main strategies, there are hybrids, such as the mosaic (Roush, 1989), which applies multiple toxins to different areas, and combination with other pest management tactics, such as biological and cultural controls (Phillips, Graves, & Luttrell, 1989). With this plethora of possible management strategies, a theory that integrates them would be beneficial for all stakeholders, including theoretical and empirical ecologists, field practitioners, and farmers. Such a theory could allow ready comparison of the effectiveness of alternative strategies, facilitating the decision making and the development of policy. With the acceleration of the rate of resistance evolution and the withdrawal of toxins, and the deceleration of discovery of new toxins (Bielza et al., 2008), it is urgent to improve resistance management and to know when and how we should use each strategy. Particularly, we need to evaluate the relative advantage among the strategies for use of multiple toxins, that is, rotation or mixture, to prolong their life spans.

Recently, the REX Consortium reviewed 16 published theoretical papers and found that the mixture strategy was superior to the rotation strategy in 14 cases, with one case the opposite and another indeterminant (REX Consortium, 2013). On the other hand, the majority of empirical researchers are still skeptical about the mixture strategy (IRAC, 2012). This is partly because rotation intuitively sounds better than mixture; mixture intensifies the selection pressure, while rotation relaxes it (Denholm & Rowland, 1992). We suspect that empirical researchers have a fundamental distrust of the simple assumptions in many theoretical models. The problem can be summarized as follows: (i) The many insect pest targets have a diversity of life histories especially in their modes and timing of toxin susceptibility and interpatch migration (Johnson, 1969), which can extend or contract the waiting time until resistance occurs, thereby influencing the optimal resistance management strategy. Nevertheless, theoretical models have often focused on single target species (but see Mani, 1989; Taylor, 1983). (ii) Both delaying resistance evolution and suppressing pest populations are essential, while many models merely report the time to resistance (but see Peck & Ellner, 1997; Peck, Gould, & Ellner, 1999).

To address these issues, we developed a simulation model that can cover various types of diploid insect pests and different insecticide application modes, for example, systemic applications and conventional spraying of insecticides. Systemic applications expose the toxin to all individuals (e.g., *Bt* crops), while conventional applications will miss some portion of the target population. Although our model assumes more realistic life histories and insecticide applications, we made its structure minimally simple following the basic formulation in the Comins model by having two patches (Comins, 1977) and similar to those used to test the efficiency of the HDR strategy for *Bt* crops (Alstad & Andow, 1995; Ives & Andow, 2002). We apply two different insecticide toxins to the pests in the simulation and query which insect type and what strategy (rotation or mixture) can effectively delay resistance evolution compared to sequential use (i.e., continuous use of one insecticide until its resistance development, then switched to another) of the toxins. We also examine the pest population size in the treated patch and show the general advantage of the mixture strategy.

## 2 MODELING

We investigate the evolution of resistance to two insecticides, designated A and B, using a spatially implicit, two‐patch model that is based on the Comins model (Comins, 1977). The model divides the landscape into two patch types: a treated patch where the insecticidal treatments are applied (patch T; proportional area 1 − *k*: The parameters used in this study are in Table 1) and a refuge where no such treatments are applied (patch N; proportional area: *k*). Although our models always have two patches, we also simulate insect life histories with no dispersal, which are equivalent to conditions of a single patch (see Section 2.1). The model assumes that the insect pest is a sexually reproducing diploid with discrete generations, and resistance to each insecticide is determined by a *R* allele at a different, unlinked locus and all other alleles at those two loci are susceptible *S* alleles. In this study, we apply the insecticides during the juvenile and/or adult stages in three different management strategies: sequential, rotation, and mixture. Through the choice of parameter values (presence/absence of three periods of insecticidal selection: juvenile, pre‐, and postmating adult), we model seven different selection regimes, which are further combined with the timing of interpatch dispersal(s) and insecticide application type (systemic or conventional spray; Table 2).

**Table 1:** State variables and parameter descriptions

Symbol	Description	Range	Default value
*p*_AT_, *p*_AN_, *p*_BT_, *p*_BN_	Frequency of the resistance allele (A/B) in the treated patch (T) and nontreated patch (N); calculated at egg stage of each generation	0 < *p* < 1	0.001 (first generation)
$n_{x,i}^{\mathrm{egg},I∼V}$	Local population density at each stage (eggs to premating adults), belonging to the genotype *x* and present in patch *i*. These state variables basically represent the density of females, but the densities of males and females are always same in this model	0 < *n*	(local carrying capacity)
$n_{x,i,j}^{\mathrm{VI}∼\mathrm{VII}}$	Local population density of postmating females, belonging to the genotype *x*, mated in patch *i*, and present at patch *j*. Only the density of females is recorded after mating (R frequency in male gametes can be retrieved from the female density in the mating patch *i*).	0 < *n*	(local carrying capacity)
*k*	Proportion of the refuge field area	0 ≤ *k* < 1	0.5
*K*	Total carrying capacity of the treated and refuge fields	0 < *K*	1.0
*r*	Fecundity, of which half is female offspring and another half is male one	1 ≤ *r*	20
*d*_pre_, *d*_post_	Dispersal rate for the pre‐ or postmating interpatch dispersal	0 ≤ *d* ≤ 1	0 (no dispersal), 1 (complete mixing)
*s*_juv_, *s*_pre_, *s*_post_	Efficacy of the insecticide application as survival probability of *SS* homozygotes	0 ≤ *s* ≤ 1	0 (systemic application), 0.1 (nonsystemic application)
*h*	Dominance as the resistance level of SR heterozygotes	0 ≤ *h* ≤ 1	0.1

**Table 2:** Types of pest life histories and pest control applications based on the presence/absence of the three selection times (juvenile, premating, and postmating adult) and interpatch dispersal times (premating and postmating adult). The presence/absence of each selection time in an insect life history depends both on the timing of the insecticide application in the pest control system and on the vulnerability of the insect to insecticide. For instance, the insects of “SD‐SM (juvenile selection–density dependence–premating selection–mating)” type are selected twice while lacking the postmating selection. Details for the timing of dispersal and selection in each insect life history are listed in Table S1

Selection type	SD‐SMS	SD‐SM	SD‐MS	SD‐M	D‐SMS	D‐SM	D‐MS
*Is the pest at the stage selected with insecticides?*							
(Juvenile)	+	+	+	+	−	−	−
(Premating)	+	+	−	−	+	+	−
(Postmating)	+	−	+	−	+	−	+
*Timing of interpatch dispersal*							
None	A brachypterous species and any pests occurring in a closed system.						
Premating	Colorado potato beetle, Many leafhoppers, Desert locust, Fig wasp		Planthopper	European pine sawfly, Diamondback moth, Codling moth, Jewel wasp, Corn earworm			
Postmating	Green rice leafhopper		Thrips, Spider mites, Phytoseiid mites, Gypsy moth	Pine shoot moth, Emerald ash borer, Bark beetle^a^			
Pre + post	Green leafhoppers, Sweet potato weevil, Asian citrus psyllid, Sunn pest		Geometer moths, Black salt marsh mosquito, Sorghum plant bug, Rice leaf bug, Capsid bug, Green bug	Many butterflies and moths, Hessian fly, Aphids^b^, Western corn rootworm, California red scale		Mediterranean fruit fly, Japanese beetle	Narrow coreid bug

a Scolytidae (partly).

b Aphids: as life cycle throughout the year, including both sexual and asexual generations.

With two loci and a resistance allele for each of the insecticidal toxins, A and B, there are nine genotypes, *S* ^A^ *S* ^A^ *S* ^B^ *S* ^B^, *S* ^A^ *R* ^A^ *S* ^B^ *S* ^B^, *R* ^A^ *R* ^A^ *S* ^B^ *S* ^B^, *S* ^A^ *S* ^A^ *S* ^B^ *R* ^B^, *S* ^A^ *R* ^A^ *S* ^B^ *R* ^B^, *R* ^A^ *R* ^A^ *S* ^B^ *R* ^B^, *S* ^A^ *S* ^A^ *R* ^B^ *R* ^B^, *S* ^A^ *R* ^A^ *R* ^B^ *R* ^B^, and *R* ^A^ *R* ^A^ *R* ^B^ *R* ^B^, where *S* is a susceptible allele and *R* is a resistance allele. Let *G* be the set of those nine genotypes. We assume that the loci are unlinked and autosomal with Mendelian inheritance. The model uses the density of the nine genotypes in the two patches as state variables, so resistance allele frequencies, *p*_AT_, *p*_AN_, *p*_BT_, and *p*_BN_, are calculated from these densities (T = treated patch; N = nontreated patch).

**Figure 1:**
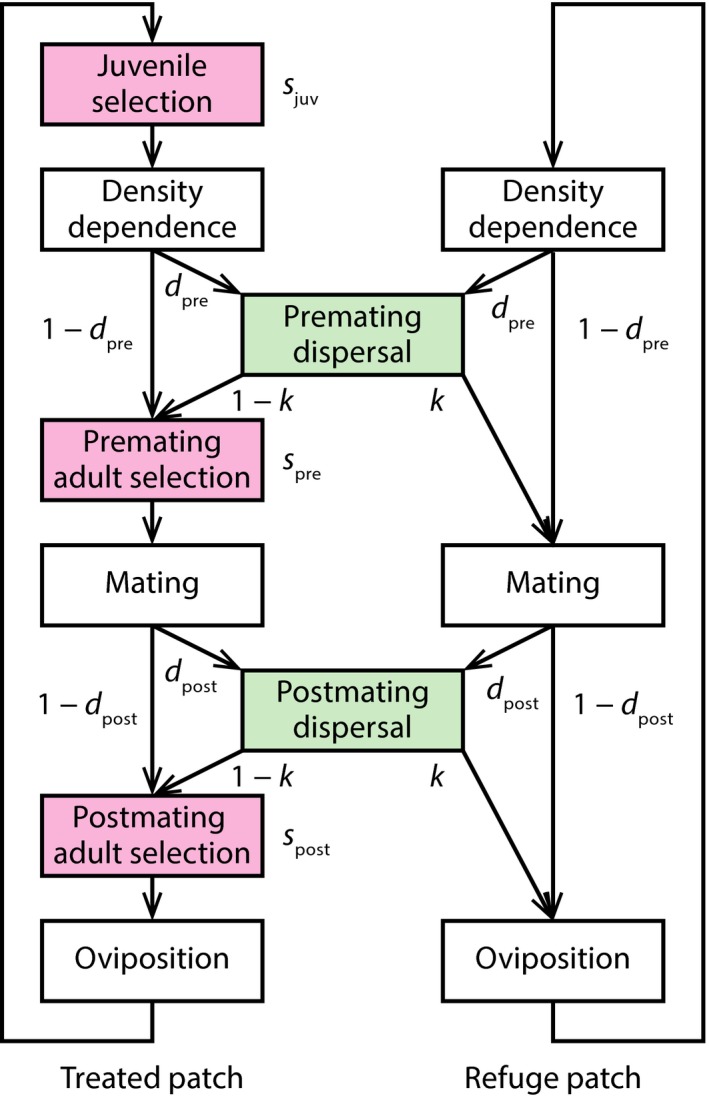
Schematic diagram of the model, showing the life‐history events in the timeline

The insect has two developmental stages: “juvenile” and “adult.” Juveniles include the egg and pupal stages, if present, and only adults have the ability to disperse between the patches. In the general model, populations pass through eight events, sequentially: (I) juvenile selection, (II) density‐dependent survival, (III) premating dispersal, (IV) premating selection, (V) mating, (VI) postmating dispersal, (VII) postmating selection, and (VIII) oviposition (Figure 1). Events I and II are during the juvenile stage and III to VIII are during the adult stage. We can classify insect life‐history types according to the presence or absence of these dispersal and selection events (Table 2).

### 2.1 Insect life histories

#### 2.1.1 Juvenile selection

In the treated patch (patch T), selection is applied to the juveniles immediately after hatching. Let $n_{x,i}^{\mathrm{egg}}\left(\tau \right)$ and $n_{x,i}^{I}\left(\tau \right)$ be an egg density of genotype *x* ∊ *G* at patch *i* ∊ {T, N} at the τ‐th generation and the corresponding density after the first step of their life cycle, that is, juvenile selection, respectively. Both state variables contain the population of males and females at a 1:1 ratio, and the sex is assumed to have the same selection survival, density dependence, and interpatch dispersals. The selection step is defined as,

(1) $$n_{x,T}^{I}\left(\tau \right)=\left\{\begin{array}{cc} \left\{s_{\mathrm{juv}}+\left(1-s_{\mathrm{juv}}\right)w_{x,A}^{\mathrm{juv}}\right\}n_{x,T}^{\mathrm{egg}}\left(\tau \right), & \left(\text{application of insecticide A}\right)\\ \left\{s_{\mathrm{juv}}+\left(1-s_{\mathrm{juv}}\right)w_{x,B}^{\mathrm{juv}}\right\}n_{x,T}^{\mathrm{egg}}\left(\tau \right), & \left(\text{application of insecticide B}\right)\\ \left\{s_{\mathrm{juv}}+\left(1-s_{\mathrm{juv}}\right)w_{x,A}^{\mathrm{juv}}w_{x,B}^{\mathrm{juv}}\right\}n_{x,T}^{\mathrm{egg}}\left(\tau \right), & \left(\text{application of mixed insecticide}\right) \end{array}\right.$$

where $w_{x,A}^{\mathrm{juv}}$, the selection survival of juveniles to insecticide A, is defined as 0, *h*, and 1 for each of the genotypes *S* ^A^ *S* ^A^, *S* ^A^ *R* ^A^, and *R* ^A^ *R* ^A^, respectively. For model simplicity, we assume insecticides A and B share the same value of dominance, *h* (i.e., $w_{x,B}^{\mathrm{juv}}$ for insecticide B is also 0, *h*, or 1 for *S* ^B^ *S* ^B^, *S* ^B^ *R* ^B^, or *R* ^B^ *R* ^B^, respectively). We note that there is no selection mortality in the refuge patch; therefore, $n_{x,N}^{I}\left(\tau \right)=n_{x,N}^{\mathrm{egg}}\left(\tau \right)$.

The *SS* survival, *s*_juv_, includes both the effects of low‐efficacy application and incomplete exposure of the insect to insecticides. As incomplete exposure is known to affect the rate of resistance evolution (Mani, 1989; Peck & Ellner, 1997), systemic and nonsystemic insecticides are modeled through *s*_juv_. When exposed to typical systemic insecticides (e.g., when applied to seedlings prior to transplanting), all individuals in a population can be exposed when they feed on the crop and *s*_juv_ is considered to be low, or almost 0. However, for nonsystemic insecticides, for example, many of which are applied by aerial application, a certain proportion of population is not exposed (0 < *s*_juv_ ≤ 1, default: *s*_juv_ = 0.1). If a target insect at the given stage is not vulnerable to the insecticide(s), *s*_juv_ is set to be 1 (i.e., the selection is absent). Following the concept of *Bt* toxin pyramiding, we modeled the mixture strategy in the present study as the exposure to an insecticide product containing both of the active ingredients, A and B, at once. Accordingly, the *SS* survival is denoted as *s*_juv_ for both single insecticide and mixture applications.

#### 2.1.2 Density‐dependent survival

We used the Beverton–Holt kernel to model density‐dependent survival, which occurs before adult emergence. Let *K*_N_ = *kK* and *K*_T_ = (1 − *k*)*K* be the carrying capacity of the refuge and the treated patch, respectively, which are assumed to be proportional to the patch area. Then, the density of emerged adults of genotype *x* in the patch *i* at τ‐th generation is defined as,

(2) $$n_{x,i}^{\mathrm{II}}\left(\tau \right)=\frac{n_{x,i}^{I}\left(\tau \right)}{1+\sum_{y\in G}n_{y,i}^{I}\left(\tau \right)/K_{i}}$$

#### 2.1.3 Premating dispersal

Premating dispersal is related to mate‐finding behavior. After adult emergence, a proportion *d* ^pre^ of the individuals in each patch leave their natal patch. These dispersers mix randomly and land back in one of the patches with a probability equal to the proportional area of the patch, that is, for a genotype *x*,

(3) $$\begin{array}{cc} & n_{x,T}^{\mathrm{III}}\left(\tau \right)=\left(1-d_{\mathrm{pre}}k\right)n_{x,T}^{\mathrm{II}}\left(\tau \right)+d_{\mathrm{pre}}\left(1-k\right)n_{x,N}^{\mathrm{II}}\left(\tau \right),\\ & n_{x,N}^{\mathrm{III}}\left(\tau \right)=d_{\mathrm{pre}}kn_{x,T}^{\mathrm{II}}\left(\tau \right)+\left(1-d_{\mathrm{pre}}+d_{\mathrm{pre}}k\right)n_{x,N}^{\mathrm{II}}\left(\tau \right). \end{array}$$

We assume that patch leaving is not density dependent. If it were an increasing function of density, the evolution of resistance would be delayed more than reported here, as the net flow from refuge to treated patch would be higher.

#### 2.1.4 Premating selection

Some insects forage on the crop after dispersal and before mating. This behavior could cause additional exposure to the pesticide. We incorporated this premating adult selection as,

(4) $$n_{x,T}^{\mathrm{IV}}\left(\tau \right)=\left\{\begin{array}{cc} \left\{s_{\mathrm{pre}}+\left(1-s_{\mathrm{pre}}\right)w_{x,A}^{\mathrm{pre}}\right\}n_{x,T}^{\mathrm{III}}\left(\tau \right), & \left(\text{application of insecticide A}\right)\\ \left\{s_{\mathrm{pre}}+\left(1-s_{\mathrm{pre}}\right)w_{x,B}^{\mathrm{pre}}\right\}n_{x,T}^{\mathrm{III}}\left(\tau \right), & \left(\text{application of insecticide B}\right)\\ \left\{s_{\mathrm{pre}}+\left(1-s_{\mathrm{pre}}\right)w_{x,A}^{\mathrm{pre}}w_{x,B}^{\mathrm{pre}}\right\}n_{x,T}^{\mathrm{III}}\left(\tau \right), & \left(\text{application of mixed insecticide}\right) \end{array}\right.$$

in the same manner as juvenile selection but with selection survival, 0, *h*, and 1 for susceptible homozygotes, heterozygotes, and resistant homozygotes, respectively (we assume the same efficacy and dominance for both pesticides). For simplicity, we also assume that dominance is the same as in the juvenile stage. Selection occurs only on the treated patch, that is, $n_{x,N}^{\mathrm{IV}}\left(\tau \right)=n_{x,N}^{\mathrm{III}}\left(\tau \right).$

#### 2.1.5 Mating

We assume random mating within a patch, so that eggs in a female are fertilized by males that originated in or moved to the patch. We assume that the genotype distribution of the males is the same as that of females in a patch and that sperm is unlimited. This step does not change population size, that is, $n_{x,i}^{V}\left(\tau \right)=n_{x,i}^{\mathrm{IV}}\left(\tau \right)$.

#### 2.1.6 Postmating dispersal

Postmating dispersal is modeled differently from premating dispersal because females are carrying male genotype information, which can differ in the two patches. Therefore, females should be tracked not only by their genotype and present location, but also by their mating patch. Let $n_{x,i,j}^{\mathrm{VI}}\left(\tau \right)$ be a density of females with genotype *x*, which mated in patch *i* and are present in patch *j* after the migration. For each female with genotype *x*, we define $n_{x,i,j}^{\mathrm{VI}}\left(\tau \right)$ as,

$$\begin{array}{cc} & n_{x,T,T}^{\mathrm{VI}}\left(\tau \right)=\left(1-d_{\mathrm{post}}k\right)n_{x,T}^{V}\left(\tau \right),\\ & n_{x,T,N}^{\mathrm{VI}}\left(\tau \right)=d_{\mathrm{post}}kn_{x,T}^{V}\left(\tau \right), \end{array}$$

and

(5) $$\begin{array}{cc} & n_{x,N,T}^{\mathrm{VI}}\left(\tau \right)=d_{\mathrm{post}}\left(1-k\right)n_{x,N}^{V}\left(\tau \right),\\ & n_{x,N,N}^{\mathrm{VI}}\left(\tau \right)=\left(1-d_{\mathrm{post}}+d_{\mathrm{post}}k\right)n_{x,N}^{V}\left(\tau \right), \end{array}$$

for females dispersing from the treated patch (T) and the refuge (N), respectively. The parameter *d*_post_ is a proportion of females that disperse after mating.

#### 2.1.7 Postmating selection

To mature eggs, some insects forage on the crop after mating, resulting in an additional exposure to the pesticides. Postmating selection is defined the same way as Equation (1),

(6) $$n_{x,i,T}^{\mathrm{VII}}\left(\tau \right)=\left\{\begin{array}{cc} \left\{s_{\mathrm{post}}+\left(1-s_{\mathrm{post}}\right)w_{x,A}^{\mathrm{post}}\right\}n_{x,i,T}^{\mathrm{VI}}\left(\tau \right), & \left(\text{application of insecticide A}\right)\\ \left\{s_{\mathrm{post}}+\left(1-s_{\mathrm{post}}\right)w_{x,B}^{\mathrm{post}}\right\}n_{x,i,T}^{\mathrm{VI}}\left(\tau \right), & \left(\text{application of insecticide B}\right)\\ \left\{s_{\mathrm{post}}+\left(1-s_{\mathrm{post}}\right)w_{x,A}^{\mathrm{post}}w_{x,B}^{\mathrm{post}}\right\}n_{x,i,T}^{\mathrm{VI}}\left(\tau \right), & \left(\text{application of mixed insecticide}\right) \end{array}\right.$$

where *i* ∊ {T, N} denotes the mating patch of females, which does not affect postmating selection survival, and in the same manner as juvenile selection, selection survival is 0, *h*, and 1 for susceptible homozygotes, heterozygotes, and resistant homozygotes, respectively (we assume the same efficacy and dominance for both pesticides). For simplicity, we also assume that dominance is the same as in the juvenile stage. Again, selection occurs only on the treated patch, that is, $n_{x,i,N}^{\mathrm{VII}}\left(\tau \right)=n_{x,i,N}^{\mathrm{VI}}\left(\tau \right)$.

#### 2.1.8 Oviposition

Lastly, females ($n_{x,i,j}^{\mathrm{VII}}\left(\tau \right)$) oviposit *r* eggs to complete the cycle, of which half (r/2) are female and another half are male. At this point, we take care to calculate the egg genotype frequencies based on the random mating assumption by converting the female densities into gamete frequencies, recombining the male and female gametes into egg genotype frequencies, and then calculating back to per‐genotype densities, $n_{x,i}^{\mathrm{egg}}\left(\tau +1\right)$.

(7) $$n_{x,i}^{\mathrm{egg}}\left(\tau +1\right)=r\left(\sum_{x\in G}n_{x,i,T}^{\mathrm{VII}}\left(\tau \right)B_{i,T}+\sum_{x\in G}n_{x,i,N}^{\mathrm{VII}}\left(\tau \right)B_{i,N}\right),$$

where **B**_*i*,*j*_ is the per‐genotype frequencies of eggs produced by females that mated in patch *i* and present in patch *j*, for which the details of the gametogenesis and fertilization operations are defined in Appendix S1.

### 2.2 Selection by multiple insecticides

Three resistance management strategies using multiple insecticides were considered: (i) *sequential use*: use of insecticide A until *p*_AT_ exceeds 0.5 (allele frequencies are monitored in eggs), then use insecticide B until the final breakdown (*p*_BT_ exceeds 0.5). (ii) *Mixture*: use of a mixture of the two insecticides. The application dose for each insecticide is the same as that in the first strategy (i.e., the so‐called full‐dose mixture strategy). (iii) *Rotation*: alternating use of the two insecticides, insecticide A for odd‐numbered pest generations and insecticide B for the even‐numbered generations.

### 2.3 Insect types categorized by life‐history events

We classified insects based on the presence and absence of juvenile, premating, and postmating selection, and presence and absence of premating and postmating dispersal. Because insects are exposed to the toxins by foraging, the presence and absence of those selections is regarded as a type of insect foraging behavior. We set a survival proportion of susceptible insects (*s*_juv_, *s*_pre_, and *s*_post_ for juvenile, premating, and postmating selections, respectively) to be 1 if corresponding selection is absent, and set *d*_pre_ (*d*_post_) to be 0 if premating (postmating) dispersal is absent. In total, we have 28 theoretical insect types (Table 2; Table S1 for the type classification of major insect pests with literature list). Furthermore, selection efficacies are set *s*_juv_ = *s*_pre_ = *s*_post_ = 0 or *s*_juv_ = *s*_pre_ = *s*_post_ = 0.1 when the systemic and nonsystemic insecticides are used, respectively.

### 2.4 Simulation procedure

All the simulations were executed using R version 3.1.1 (R Core Team, 2015). In the first generation, we set egg genotypes to the Hardy–Weinberg equilibrium with the low initial frequencies of the resistant alleles, *p*_A_ = *p*_B_ = 0.001, as was assumed in previous works (Gould, 1998; Gould et al., 1997; Roush, 1998). In the refuge patch, the initial insect population was set to the carrying capacity, *kK*. Although the corresponding population size in the treated patch would be (1 − *k*)*K*, it was initialized to 0 except when *k* = 0 (no refuge) or *d*_pre_ = *d*_post_ = 0 (no dispersal). This simulates a population that had been completely eliminated by some other pest control measure, or establishment of new fields where the pest had not previously occurred. Thus, the treated patch population originates from the refuge patch. Resistance evolution was monitored until the *R* ^B^ allele frequency in the treated patch exceeded 0.5 or the number of generations exceeded 100,000.

In addition, the composition of the local mating pool (*p*_AT_ and *p*_BT_) and the population density of the pest insects within the treated patch were monitored for each of the parameter sets used in this study. The frequencies were measured just before mating and the population densities were measured at adult emergence of the fourth generation. Population densities had reached a quasi‐equilibrium by the fourth generation.

## 3 RESULTS

### 3.1 Effect of life‐history types on resistance evolution

**Figure 2:**
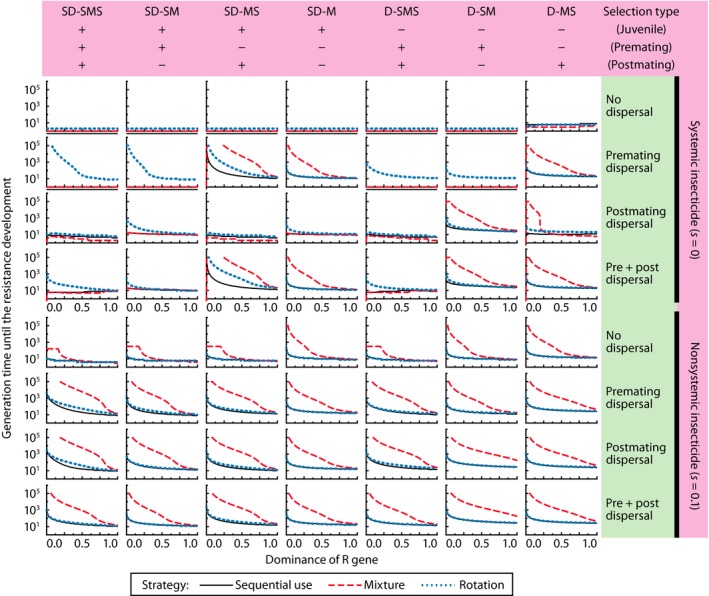
Dominance of *R* alleles, insect life histories, and insecticide application efficacy affecting the waiting time to resistance (*R* ^B^ allele frequency reaches 50% in the treated field), provided insecticides are used in sequential (black thin line), mixture (red broken), and rotation (blue dotted) strategies. Other parameters at their default values. Some values are not plotted where the waiting time is estimated >100,000 generations (the maximum of calculation)

For nonsystemic insecticide application (*s*_juv_, *s*_pre_, *s*_post_ > 0), the mixture strategy was superior to the other two in retarding resistance evolution regardless of the mode of interpatch dispersal, insect life history, or dominance (Figure 2, the fifth to eighth rows). The low efficacy allows susceptible genotypes to survive the mixture in the treated patches. The rate of resistance evolution when the proportional area of the refuge patch was 0.1 (Fig. S1) showed the same tendency as when the refuge proportion was 0.5 (Figure 2), although resistance generally evolved faster with a smaller refuge.

**Figure 3:**
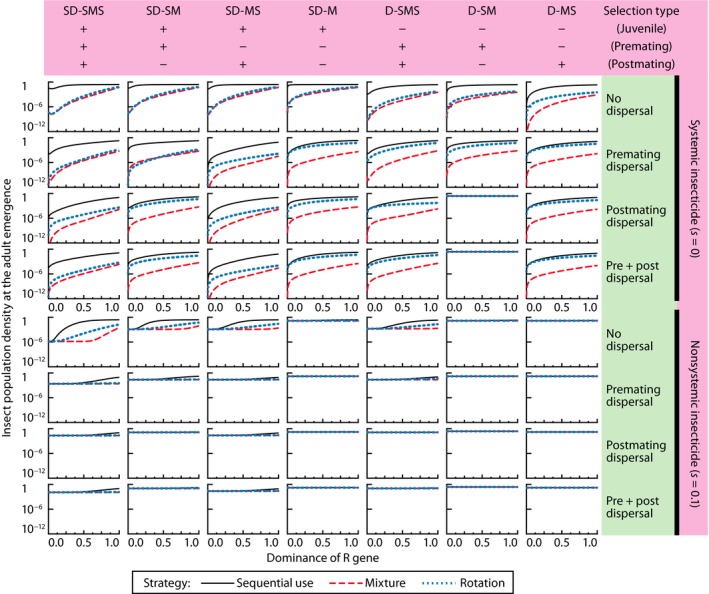
Relative insect population density (the density divided by the local carrying capacity, (1 − *k*) *K*) in the treated field at adult emergence of the fourth generation. Other parameters at their default values

For systemic insecticides, the mixture strategy was optimal when premating dispersal occurs without premating selection (Figure 2, rows 2 and 4, columns 3, 4, and 7). Premating dispersal provides susceptible adults to the treated patch, which will mate with resistant adults. The mixture strategy did not perform well for some parameter combinations, especially when premating adult selection is strong (*s*_pre_ ≅ 0; Figure 2, rows 1–4, columns 1, 2, 5, and 6). Even when there is premating dispersal, the subsequent premating selection will eliminate nearly all susceptible genotypes in the treated patch, resulting in high inbreeding of double‐resistant genotypes and rapid resistance evolution. This phenomenon was also found in numerical simulations of resistance evolution in Colorado potato beetle, *Leptinotarsa decemlineata* (Chrysomelidae), where adults mate after the intensive selection by the rotation or mixture of multiple insecticides (Argentine, Clark, & Ferro, 1994). Even so, there were two exceptions (Figure 2, rows 3 and 4, column 6), where the mixture strategy was optimal when there was premating selection. This occurred when there was postmating dispersal and no juvenile or postmating selection, and is associated with the strong density‐dependent mortality in the treated patch. With postmating dispersal, the treated patch is replenished with susceptible individuals, and without juvenile selection, the population in the treated patch reaches carrying capacity (Figure 3), reducing the absolute fitness of the resistant individuals almost to mere replacement level. Consequently, the resistant alleles no longer have a large fitness advantage, and resistance evolution is delayed.

The performance of the rotation strategy is always equal to or better than the sequential use of each insecticide, but it is optimal only under a few conditions. When systemic insecticides are used (*s* ≅ 0), the rotation strategy performs better than the mixture strategy when there is premating dispersal combined with the premating adult selection (Figure 2, rows 2 and 4, columns 1, 2, 5, and 6). This advantage stems from the alternating selection in the rotation, which allows susceptible alleles of the unselected toxin to increase in alternating generations in the treated patch. Selection in the rotation will eliminate susceptibles of only one of the toxins in the treated patch, leaving $R^{A}R^{A}S^{B}S^{B}\;\text{and}\;R^{A}R^{A}S^{B}R^{B}$ survivors to mate with the rare $R^{A}R^{A}R^{B}R^{B}$ survivors when selection is with insecticide A. Even though premating selection will eliminate susceptible individuals for one of the toxins, in this case to insecticide A, some of these insects are susceptible homozygotes to insecticide B, which will convey and multiply *S* ^B^ alleles to the offspring. The evolutionary delay will be greatest when dominance is recessive or partially recessive (*h* < 0.5), as resistance alleles are eliminated as heterozygotes. As the efficacy of the premating adult selection declines, however, the advantage of the rotation strategy disappears (Figure 2: for rows 2 and 4, compare columns 1 and 3, 2 and 4, and 5 and 7). This is because the mixture strategy improves substantially as more susceptible individuals survive selection.

Without premating dispersal, there is one more condition where the rotation strategy is more advantageous than the mixture and sequential use strategies. This occurs for recessive and partially recessive resistance when the population has only postmating dispersal and strong juvenile selection, lacking postmating adult selection (Figure 2, row 3, columns 2 and 4). In this case, the $S^{A}S^{A}R^{B}R^{B}$ and $S^{A}R^{A}R^{B}R^{B}$ immigrants survive selection by the B insecticide and increase in the next generation, thereby delaying resistance evolution.

### 3.2 Effect of the dominance of R genes

Resistance generally evolved more slowly when dominance was low, except for the life histories without interpatch dispersal with systemic insecticides (Figure 2, row 1). The mixture and/or rotation strategies perform better than sequential use as long as most of the mating pool in the treated field is susceptible alleles (Fig. S2). These results suggest that the mechanisms underlying the HDR strategy are the main mechanisms that delay resistance evolution when multiple insecticides are used.

Although most of the known resistance alleles to *Bt* crops are incompletely recessive (Bourguet, Genissel, & Raymond, 2000), dominant resistance is also known in *Bt* cotton (Carrière, Crowder, & Tabashnik, 2010; Jin et al., 2013). For conventional insecticides, resistance caused by target site mutation varies from completely recessive to completely dominant, although mutations in ion channels and receptor targets are in general completely or incompletely recessive (Bourguet & Raymond, 1998). Therefore, an optimal management strategy must be robust to high dominance. Our results show that the requirement for recessive resistance in the high‐dose strategy can be relaxed when using mixtures. A “moderate dose,” with dominance 0.2 < *h* < 0.8, for both insecticides in a mixture can kill heterozygotes at the same rate as a single high‐dose insecticide, thereby achieving equivalent or even longer waiting times to resistance (Figure 2). This effect was already reported in the simulations of Roush (1998), but our results show that it holds more generally for various insect life histories. In contrast, the rotation strategy required a higher dose (lower dominance) to achieve the same waiting time as the mixture strategy at a lower dose.

### 3.3 Effects of interpatch dispersal and selection efficacy

The refuge patch serves as a source of susceptible alleles, and the parameter *d*, dispersal rates of insects, corresponds to the supply rate in our models. In most cases, resistance evolves more slowly at *d* = 1 than at *d* = 0 (Figure 2). On the other hand, if selection comes before mating, insecticide application reduces the supply of susceptible alleles, thereby increasing the rate of evolution (compare Figure 2, rows 2 and 6). Therefore, low‐efficacy selection (large *s*) retards the resistance evolution, as long as high dosage (low dominance) is ensured.

When enough of the juvenile population is left intact in the treated patch, the adult population density in the treated patch may increase up to nearly the carrying capacity in the next generation (the nonsystemic insecticide application in Figure 3). Overall, the population density of adult insects emerging in the treated area depends largely on *s* and *h*, the efficacy and the dominance of the selection. If even a small number of insects reduce the commercial value of the products, such as some ornamental plants, vegetables, and fruits, we need to achieve nearly perfect control of the pests (*s* ≅ 0) using systemic insecticide applications, high‐dose *Bt* crops, or well‐targeted and/or frequent use of nonsystemic insecticides. In most cases where systemic insecticides are used, the mixture strategy outperforms the rotation and sequential use strategies in controlling the insect density (Figure 3).

As Comins (1977) implied, when the nonsystemic insecticides are used (*s*_juv_, *s*_pre_, *s*_post_ > 0), survivors in the treated field also serve as a reservoir of *S* alleles and will retard resistance evolution, even when there is no external refuge patch (Figure 2, row 5). This effect is particularly prominent when two high‐dose active ingredients are applied in a mixture. So one might believe that refuges are not needed for nonsystemic, high‐dose pesticides applied in mixture. In practice, however, the population density will be high (Figure 3) and it will be difficult to balance the conflicting demands or retarding resistance and controlling the population. Nevertheless, the mixture strategy for nonsystemic insecticides may work well because of its robustness to non‐high‐dose (higher dominance, with *h* < 0.5).

### 3.4 Effect of refuge proportion

**Figure 4:**
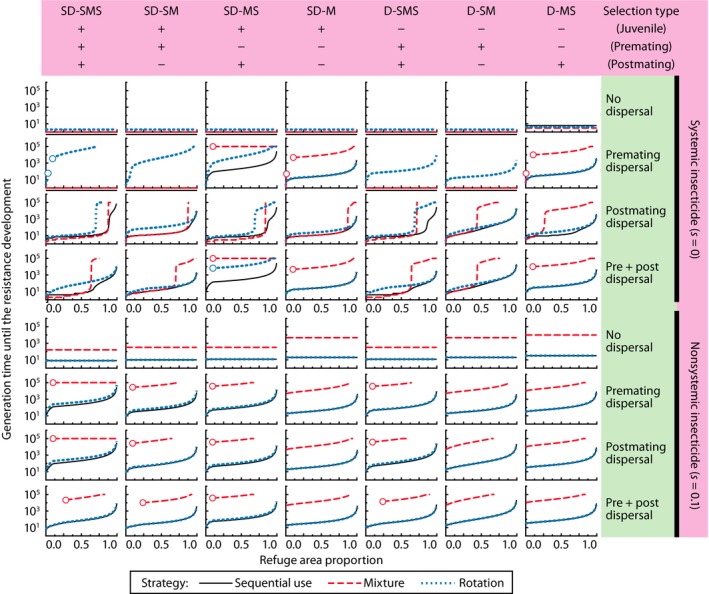
Effect of the proportional size of the refuge patch (*k*) on the waiting time to resistance. Some values are not plotted for small *k* (endpoints with open circles) because of the local extinction of the population (population density in the treated patch reached below the limit of calculation, 10^−20^(1 − *k*) *K*, before resistance evolved). The dominance of the resistance genes, *h*, was fixed at 0.1. Other parameters at their default values

Across entire range of the refuge proportion (0 ≤ *k* < 1), both the rotation and mixture strategies have a waiting time equal to or greater than the sequential use strategy (Figure 4). While the refuge proportion and dominance of the resistance alleles strongly affect the waiting time, the mixture strategy is the optimal management strategy whenever a small portion of the susceptible population in the treated patch escapes selective mortality (Figures 4, S3, and S4). In contrast, the optimal strategy for systemic insecticides changes between the mixture and rotation strategies, depending on adult selection, dispersal, and refuge proportion (Figs [Link], [Link], [Link]). The rotation strategy is optimal when there is premating adult selection and (i) only premating adult dispersal, or (ii) postmating adult dispersal and small enough refuge proportions, or (iii) no premating adult selection and only postmating dispersal and small enough refuge proportions. There was no case where the sequential use strategy was better than the other two strategies (Fig. S3).

Some previous studies argued that pyramiding two active *Bt* toxins (mixture strategy) relaxes the requirements for both the dose level and refuge proportion [Zhao et al., 2003, 2005; but see Alyokhin (2011)]. Our results show that the required refuge proportion for the mixture strategy with systemic insecticides, such as pyramided high‐dose *Bt* toxins, depends on the life history of insects and cannot be determined generally (Figure 4, rows 2–4). It can be concluded that the “small refuge and pyramiding” strategy is compatible with the “large refuge and single toxin” only when the participation of susceptible adults in mating is ensured. When the insecticides are nonsystemic, the mixture strategy has a long waiting time regardless of the refuge proportion (Figure 4), as susceptible homozygotes persist in the treated field. For the rotation strategy, the waiting time always increases monotonically as the treated area size decreases, except for the case of “no dispersal,” in which there is no functional refuge patch.

As mentioned above, when the refuge proportion is large enough to supply susceptibles to the entire system through postmating dispersal, and juvenile selection is absent, a large number of susceptible juveniles develop in the treated patch (*s*_juv_ ≫ 0 and *d*_post_ > 0: Figure 4, rows 3–4, column 6). Subsequent density dependence will suppress the rate of population growth of resistant insects in the treated patch, which retards resistance evolution especially for the mixture strategy. Under these conditions, however, considerable yield loss will take place because the population size in both patches is high, so these scenarios will not be practical even though they have an advantage for resistance management.

## 4 DISCUSSION

There has been a long‐standing debate about whether the rotation or mixture strategy will be most effective for pesticide resistance management. Our results support the robustness of the mixture strategy for non‐high‐dose insecticides when insecticide application is imperfect or a refuge supplies susceptible insects to the mating pool and the initial resistance frequency is low. We showed this superiority using a mechanistic model applied to a variety of diploid insect life histories. Although the REX Consortium reported a similar conclusion, they merely counted the number of theoretical studies that compared the rotation and mixture strategies (REX Consortium, 2013). We found that the mixture strategy shares the same mechanism as the HDR strategy to delay resistance evolution as suggested by Ives et al. (2011).

Previous theoretical works emphasized the importance of the HDR strategy to manage resistance evolution while suppressing pest density (Alstad & Andow, 1996; Comins, 1977; Taylor & Georghiou, 1979). The HDR strategy has been quite successful, especially in the management of genetically modified, high‐dose *Bt* crops. Although resistance to *Bt* crops has been reported in the field, these were nearly all non‐high‐dose *Bt* crops, or in a few cases, lacked an adequate refuge (Campagne, Kruger, Pasquet, Le Ru, & Van den Berg, 2013; Tabashnik et al., 2013). The HDR strategy might be useful for conventional insecticides, but the implementation of a high dose is difficult. One reason is the increase in the functional dominance of an insecticide through chemical degradation and spatially variable application (Denholm & Rowland, 1992). Strategies for combining two or more active ingredients over time and space have been advocated to overcome these disadvantages (Denholm & Rowland, 1992; REX Consortium, 2013). The mixture strategy usually relaxes the necessity for a high dose (Roush, 1998; Figure 2) and may be useful for resistance management if we can neglect the additional cost of more than one insecticide and possibly increased environmental risk.

Several non‐high‐dose *Bt* crops have been used. Nearly all of these were released as single toxin *Bt* crops, and resistance in the target pest evolved quickly. For example, *Spodoptera frugiperda* (Noctuidae) evolved resistance to Cry1F *Bt* maize in only a few years in Puerto Rico (Storer et al., 2010) and Brazil (Farias et al., 2014) and has a non‐high‐dose resistance (*h* = 0.15) (Farias et al., 2016). Cry1F *Bt* maize originally acted like a systemic insecticide with very low survival, selection was only on juveniles, and it had both pre‐ and postmating dispersal (Figure 2, row 4, column 4). In this case, our results predict that a pyramided variety would last much longer than a single toxin or sequential use of single toxins. *Diabrotica virgifera* (Chrysomelidae) also evolved resistance rapidly, in this case to Cry3Bb *Bt* maize in the US Midwest (Gassmann, Petzold‐Maxwell, Keweshan, & Dunbar, 2011). Although the genetic basis of resistance is not yet known, resistance alleles may be codominant or only slightly recessive (*h* ≤ 0.5). Additionally, all of the *Bt* maize varieties active against this species act as nonsystemic insecticides, juvenile selection is strong, pre‐ and postmating selection is probably weak, and most dispersal is postmating (Andow et al., 2016; Figure 2, row 7, column 4 and possibly columns 1–3). Our results predict that a pyramided variety would last much longer than sequential use or rotation of single toxins and that this prediction is robust to the presence or absence of pre‐ and postmating selection.

In several situations of systemic insecticides or *Bt* crops, however, there are cases where the mixture strategy is not the best one (Figure 2). Of the 28 insect life histories (Table 2), there were three outcomes: (i) The mixture strategy best delays resistance evolution, (ii) the rotation strategy best delays resistance evolution, and (iii) no strategy delays resistance evolution. The insect type with “juvenile‐only selection (SD‐M) with premating dispersal” (Figure 2, rows 2 and 4, column 4) is an example of case (i). Many Lepidopteran species may belong to this insect type (Tables 2, S1) and includes several of the main target species of *Bt* cotton and *Bt* maize, such as tobacco budworm (*Heliothis virescens*, Noctuidae), pink bollworm (*Pectinophora gossypiella*, Gelechiidae), European corn borer (*Ostrinia nubilalis*, Crambidae), and southwestern corn borer (*Diatraea grandiosella*, Crambidae) (Table S1). The insect type with “juvenile and postmating adult selection (SD‐MS) with premating dispersal” (Figure 2, rows 2 and 4, column 3) is another case (i). This type includes the major hemipteran pests of rice fields in East Asia. A mixture of systemic insecticides, imidacloprid and fipronil, is already sold for planthopper control. A mixture of systemic insecticides is a sound resistance management strategy for these insects as long as resistance is not too dominant (Figure 2). However, care must be taken if pests have been selected for a single component of the mixture as adaptation to the single component may be a stepping stone to resistance to the mixture (equivalent to the sequential strategy). For example, the small brown planthopper, *Laodelphax striatellus* (Delphacidae), already has some resistance to one of the components of the mixture in Japan (Sanada‐Morimura et al., 2011).

For case (ii), the insect type with “juvenile, pre‐ and postmating adult selection” (SD‐SMS, Figure 2, column 1) should not be managed with the mixture strategy. If there is the premating dispersal (Figure 2, rows 2 and 4), rotation of systemic insecticides when *h* < 0.5 can effectively retard resistance evolution. A well‐known example is Colorado potato beetle. Many conventional chemical insecticides, including systemic ones, have been lost to beetle resistance, some because of high dominance (Alyokhin, Baker, Mota‐Sanchez, Dively, & Grafius, 2008; Alyokhin et al., 2015). Our results suggest that rotating insecticides should be considered to combat rapid resistance evolution in this beetle (Alyokhin et al., 2015). However, the advantage of rotation disappears with an increase in *r*, the reproduction rate of the insect population, so the rotation strategy should be used with care (see Appendix S2, Figure S9).

For case (iii), there is no resistance management strategy available for insect types with “juvenile‐only selection (SD‐M) without premating dispersal” (Figure 2, rows 1 and 3, column 4) and “juvenile and postmating adult selection (SD‐MS) without premating dispersal” (Figure 2, rows 1 and 3, columns 1 and 3). The former class probably contains important forest pest insects, for example, the European pine shoot moth, *Rhyacionia buoliana* (Tortricidae), and the Emerald ash borer, *Agrilus planipennis* (Buprestidae; Table S1). The latter class probably contains the minute but serious pests of thrips, for example, the western flower thrips, *Frankliniella occidentalis* (Thripidae), and spider mites, for example, *Tetranychus urticae* (Tetranychidae). Multiple resistance is already widespread in those groups (Bielza, 2008; van Leeuwen, Vontas, Tsagkarakou, Dermauw, & Tirry, 2010). Pest management that does not rely on chemical pesticides, for example, mechanical and cultural control and/or biological control, may be required for such the pests.

Some genetic and physiological factors can reduce the advantage of mixtures over the other management strategies. First is cross‐resistance (Argentine et al., 1994; Roush & McKenzie, 1987). As the mechanism of cross‐resistance is often idiosyncratic and difficult to generalize, we examined a simple variant model that assumed reciprocal cross‐resistance between the two *R* alleles (an individual having a *R* ^A^ allele has some survival under exposure to insecticide B, and vice versa). The result was, in brief, that the advantage of the mixture over rotation decreased as the degree of cross‐resistance increased, but rarely the mixture was less than the rotation strategy even under complete cross‐resistance (Fig. S6).

Second, linkage between the resistance loci would decrease the waiting time in the mixture strategy. If there is a positive linkage disequilibrium, that is, the frequency of *R* ^A^ *R* ^B^ gamete is larger than the product, *p*_A_ *p*_B_, it will reduce the performance of the mixture (Mani, 1985). Although the effect of initial linkage disequilibrium rapidly decreases and scarcely affects the total waiting time when the recombination rate is larger than zero (Mani, 1985), strong selection will maintain positive linkage disequilibrium (Crow & Kimura, 1970) reducing, but probably not eliminating the advantage of the mixture over the rotation strategy.

Third, if the resistance allele has a fitness cost, rotation and sequential use may become more advantageous than the mixture strategy (Curtis, Hill, & Kasim, 1993; Immaraju, Morse, & Hobza, 1990). We preliminarily evaluated the fitness cost using another variant of our model assuming that a resistant insect has a lower egg hatching success. To model a fitness cost of resistance to insecticide B, we incorporated a cost in egg hatching success with the following operation before juvenile selection:

$$n_{x,i}^{{\mathrm{egg}}^{\prime }}\left(\tau \right)=\left\{\begin{array}{cc} \zeta_{\mathrm{susB}}n_{x,i}^{\mathrm{egg}}\left(\tau \right), & \left(\;\text{for}\;S^{B}S^{B}\right)\\ \zeta_{\mathrm{heteroB}}n_{x,i}^{\mathrm{egg}}\left(\tau \right), & \left(\;\text{for}\;S^{B}R^{B}\right)\\ \zeta_{\mathrm{resB}}n_{x,i}^{\mathrm{egg}}\left(\tau \right), & (\;\text{for}\;R^{B}R^{B}). \end{array}\right.$$

The coefficients ζ_susB_, ζ_heteroB,_ and ζ_resB_ are the egg hatching success of the susceptible *S* ^B^ *S* ^B^ homozygotes, *S* ^B^ *R* ^B^ heterozygotes, and the resistant *R* ^B^ *R* ^B^ homozygotes, respectively. Then, $n_{x,i}^{{\mathrm{egg}}^{\prime }}\left(\tau \right)$ is substituted into the equation of juvenile selection for $n_{x,i}^{\mathrm{egg}}\left(\tau \right)$.

The waiting time until resistance increased as a larger fitness cost was imposed on the *R* ^B^ *R* ^B^ homozygotes (Fig. S7). When a 0.9 relative fitness was imposed on the resistant homozygote of insecticide B (ζ_resB_ = 0.9, while ζ_susB_ = ζ_heteroB_ = 1.0), the waiting time until resistance increased in both the mixture and rotation strategies. In the sequential use strategy, the *R* ^B^ allele was eliminated from the population before fixation of *R* ^A^, so for this strategy the total generation time before resistance breakdown could not be determined (Fig. S8). When heterozygotes also have an intermediate fitness cost (ζ_susB_ = 1.0, ζ_heteroB_ = 0.95,  and ζ_resB_ = 0.9), the extinction of *R* ^B^ haplotype occurred in all strategies. Consequently, it was not possible to determine the optimal management strategy in the presence of fitness costs.

Although this work provides a theoretical basis for understanding the optimal resistance management strategy for two toxins, in specific applications, it may be necessary to relax some of the assumptions inherent in the two‐patch mechanistic model we used. For example, a spatially explicit model may be required when dispersal distances must be considered, or a stochastic model may be necessary to evaluate the effects of finite population size. In addition for specific cases, particular dispersal (0 < *d*_*x*_ < 1) and refuge values (*k* ≠ 0.5) should be examined, as the waiting time to resistance is a nonlinear function of these parameters (Takahashi, Yamanaka, Sudo, & Andow, 2017).

To conclude, we found that the optimal resistance management strategy should take account (i) whether or not insects disperse between the exposure to insecticides and mating and (ii) whether the pest control program keeps the density of target insects nearly zero (i.e., systemic control). If we can tolerate a small but substantial susceptible population in the treated patch, the mixture strategy will extend the life span of insecticides without requiring high dose or any specific insect life history. However, if it is necessary to kill almost all the susceptible individuals by using a high‐efficacy pesticide, such as a systemic insecticide, the mixture strategy is optimal only when there is interpatch dispersal just before mating. Otherwise, only the rotation strategy can retard the resistance evolution longer than the sequential strategy. The relationship among the insecticidal dose (effective dominance), the efficacy of the application (i.e., survival rate of susceptible insects), and the subsequent injury level on the crop needs additional investigation to maximize the effectiveness of those resistance management strategies within a pest management framework for agriculture.

## DATA ARCHIVING

R source code used is from the Dryad Digital Repository: https://doi.org/10.5061/dryad.4gv44.
