# Supplementary material for: Optimal management strategy of insecticide resistance under various insect life histories: Heterogeneous timing of selection and interpatch dispersal
Source: Evol Appl. 2017 Nov 2;11(2):271–83. doi: 10.1111/eva.12550 (PMC5775500; doi:10.1111/eva.12550)
Supplement: Supplementary file 10 [file EVA-11-271-s010.docx]

# Electronic supplementary materials

Supplementary Table S1. Literature list for the insect type-classification based on the phase of dispersal and the timings of possible insecticide application in the life-cycle. Including some non-pest insects.

| Pest insect | | | Timing of dispersal | Possible timing for selection | Remarks | Literature |
| --- | --- | --- | --- | --- | --- | --- |
| Order | (Super-) Family | Species (location) |  |  |  |  |
| (Options) | | | (pre-mating, post-mating) | (juvenile, pre-mating, post-mating) |  |  |
| Diptera | Culicidae | *Aedes taeniorhynchus* | pre (female) + post | juvenile + (pre) + post | Black salt marsh mosquito | [1] |
|  | Cecidomyiidae | *Mayetiola destructor* | pre (male) + post | juvenile | Hessian fly | [2] |
|  | Tephritidae | *Ceratitis capitata* | pre (male/female) + post | pre | Mediterranean fruit fly: when conventional insecticide is used. | [3] |
| Coleoptera | Chrysomelidae | *Leptinotarsa decemlineata*  (in the United States) | pre (male/female) | juvenile + pre + post | Colorado potato beetle: multiple mating | [4,5] |
|  |  | *Diabrotica virgifera* | pre (male) + post | juvenile (+ pre + post) | Western corn rootworm (selection pressure on adult is uncertain) | [6] |
|  | Brentidae | *Cylas formicarius* | pre (male/female?) + post? | juvenile + pre + post | Sweet potato weevil: female mobility is questionable | [7,8] |
|  | Buprestidae | *Agrilus planipennis* | post | juvenile | Emerald ash borer | [9,10] |
|  | Scarabaeidae | *Popillia japonica* | pre (male/female) + post | (juvenile) + pre | Japanese beetle | [11] |
| Hemiptera | Aphidoidea |  | pre (male and/or female) + post | juvenile | Aphids: as a life-cycle throughout the year, including both sexual and asexual generations (i.e. juvenile = asexual morph). | [12,13] |
|  | Cicadellidae | *Nephotettix cincticeps* | post | juvenile + pre + post | Green rice leafhopper | [14–16] |
|  |  | *Cicadella viridis* | pre + post | juvenile + pre + post | Green leafhoppers | [15] |
|  |  | *Laburrus impictifrons* | pre | juvenile + pre + post | Leafhopper | [15] |
|  | Delphacidae |  | pre (female) | post | Planthopper: long-range (over-sea) dispersal is not included. | [15,17–19] |
|  | Diaspididae (Coccoidea) | *Aonidiella aurantii* | pre (male) + post (as juvenile) | juvenile | California red scale: dispersal of juvenile is included in the post-mating dispersal. | [20] |
|  | Psyllidae | *Diaphorina citri* | pre (male/female) + post | juvenile + pre + post | Asian citrus psyllid | [21] |
|  | Scutelleridae | *Eurygaster integriceps* | pre (male/female) + post | juvenile + pre + post | Sunn pest: including long-range migration | [22] |
| Heteroptera | Coreidae | *Cletus punctiger* | pre (male/female) + post | post | Narrow coreid bug | [23] |
|  | Miridae | *Stenotus rubrovittatus* | pre (male/female) + post | juvenile + post | The sorghum plant bug: multiple mating | [25] |
|  |  | *Trigonotylus caelestialium* | pre (male/female) + post | juvenile + post | The rice leaf bug: multiple mating | [24] |
| Hymenoptera | Chalcidoidea |  | pre (male) | juvenile + pre + post | Fig wasp | [26] |
|  | Diprionidae | *Neodiprion sertifer* | pre (male) | juvenile | European pine sawfly | [27] |
|  | Pteromalidae | *Nasonia vitripennis* | pre (female) + post | juvenile | Jewel wasp | [28,29] |
| Lepidoptera |  |  | pre (male/female) + post | juvenile | Many species | [22] |
|  | Crambidae | *Diatraea grandiosella.* | pre (male/female?) + post | juvenile | Southwestern corn borer: pre-mating female dispersal is questionable. | [30] |
|  | Crambidae | *Ostrinia nubilalis* | pre (male/female?) + post | juvenile | European corn borer: pre-mating female dispersal is controversy. | [31–33] |
|  | Geometridae | *Operophtera brumata* | pre (male) + post (as larva) | juvenile + post (as larva) | Winter moth: wingless adult female | [34,35] |
|  | Hesperidae | *Hylephila phylaeus* | pre (male/female) + post | juvenile | Skipper | [36] |
|  | Noctuidae | *Helicoverpa zea* | pre (male/female) | juvenile | Corn earworm | [37] |
|  |  | *Spodoptera litura* | pre (male/female) | juvenile | Oriental leafworm moth | [38,39] |
|  | Pieridae | *Pieris rapae* (in Japan) | pre (female) + post | juvenile | Small white | [40] |
|  |  | *Pieris rapae* (in the United States) | pre (male) + post | juvenile |  | [41] |
|  | Pyralidae | *Cnaphalocrocis medinalis* | pre (male/female) + post | juvenile | Rice leafroller | [42] |
|  | Plutellidae | *Plutella xylostella* | pre (male) | juvenile | Diamondback moth | [43] |
|  | Tortricidae | *Rhyacionia buoliana* | post | juvenile | European Pine Shoot Moth | [22,44] |
|  | Lymantriidae | *Lymantria dispar dispar* | post (as larva) | juvenile + post (as larva) | Gypsy moth: wingless adult female. | [45] |
|  | Tortricidae | *Cydia pomonella* | pre (male/female) | juvenile | Codling moth | [46] |
| Orthoptera | Acrididae | *Schistocerca gregaria* | pre (male/female) | juvenile + pre + post | Desert Locust | [22,47,48] |
| Thysanoptera |  |  | post (female) | juvenile + post | Thrips: in the cases of arrhenotoky | [49,50] |
| Acari | Tetranychidae | *Tetranychus urticae* | post | juvenile + post | Two-spotted spider mite: aerial dispersal | [51] |
|  | Phytoseiidae |  | post | juvenile + post | Phytoseiid mites: aerial dispersal. | [52] |

## References

1. Provost, M. W. 1952 The dispersal of *Aedes taeniorhynchus*. I. Preliminary studies. *Mosq. News* **12**, 174–190.

2. Harris, M. O., Stuart, J. J., Mohan, M., Nair, S., Lamb, R. J. & Rohfritsch, O. 2003 Grasses and gall midges: plant defense and insect adaptation. *Annu. Rev. Entomol.* **48**, 549–577.

3. Hendrichs, J., Katsoyannos, B. I., Papaj, D. R. & Prokopy, R. J. 1991 Sex differences in movement between natural feeding and mating sites and tradeoffs between food consumption, mating success and predator evasion in Mediterranean fruit flies (Diptera: Tephritidae). *Oecologia* **86**, 223–231.

4. Alyokhin, A. V. & Ferro, D. N. 1999 Reproduction and dispersal of summer-generation Colorado potato beetle (Coleoptera: Chrysomelidae). *Environ. Entomol.* **28**, 425–430.

5. Hare, J. D. 1990 Ecology and management of the Colorado potato beetle. *Annu. Rev. Entomol.* **35**, 81–100.

6. Marquardt, P. T. & Krupke, C. H. 2009 Dispersal and mating behavior of *Diabrotica virgifera* virgifera (Coleoptera: Chrysomelidae) in Bt cornfields. *Environ. Entomol.* **38**, 176–182.

7. Moriya, S. & Hiroyoshi, S. 1998 Flight and locomotion activity of the sweet potato weevil (Coleoptera: Brentidae) in relation to adult age, mating status, and starvation. *J. Econ. Entomol.* **91**, 439–443.

8. Sakuratani, Y., Sugimoto, T., Setoguchi, O., Kamikado, T., Kiritani, K. & Okada, T. 1994 Diurnal changes in micro-habitat usage and behavior of *Cylas formicarius* (Fabricius)(Coleoptera: Curculionidae) adults. *Appl. Entomol. Zool.* **29**, 307–315.

9. Taylor, R. A. J., Bauer, L. S., Poland, T. M. & Windell, K. N. 2010 Flight performance of *Agrilus planipennis* (Coleoptera: Buprestidae) on a flight mill and in free flight. *J. Insect Behav.* **23**, 128–148.

10. Mercader, R. J., Siegert, N. W., Liebhold, A. M. & McCullough, D. G. 2011 Simulating the effectiveness of three potential management options to slow the spread of emerald ash borer (*Agrilus planipennis*) populations in localized outlier sites. *Can. J. For. Res.* **41**, 254–264.

11. van Timmerman, S. J., Switzer, P. V. & Kruse, K. C. 2001 Emergence and reproductive patterns in the Japanese beetle, *Popillia japonica* (Coleoptera: Scarabaeidae). *J. Kans. Entomol. Soc.* , 17–27.

12. Dixon, A. F. G. 1977 Aphid ecology: life cycles, polymorphism, and population regulation. *Annu. Rev. Ecol. Syst.* , 329–353.

13. Moran, N. A. 1992 The evolution of aphid life cycles. *Annu. Rev. Entomol.* **37**, 321–348.

14. Kuno, E. & Hokyo, N. 1970 Comparative analysis of the population dynamics of rice leafhoppers, *Nephotettix cincticeps* Uhler and *Nilaparvata lugens* Stål, with special reference to natural regulation of their numbers. *Res. Popul. Ecol.* **12**, 154–184.

15. Katayama, E. 1987 Ovarian development and proportion of mated individuals in plant- and leaf-hoppers collected by light traps. *Jpn. J. Appl. Entomol. Zool.* **31**, 264–266. (doi:10.1303/jjaez.31.264)

16. Katayama, E. 1975 The Relation between Ovarial Development and Copulation in a Leafhopper and Plant-hoppers of Rice Plant (Homoptera: Auchenorrhyncha). *Jpn. J. Appl. Entomol. Zool.* **19**, 176–181. (doi:10.1303/jjaez.19.176)

17. Matsumura, M. 1997 Seasonal prevalence of ovarian development in macropterous females of whitebacked planthopper, *Sogatella furcifera* (Horváth) (Hemiptera: Delphacidae) in paddy fields. *Jpn. J. Appl. Entomol. Zool.* **41**, 75–82. (doi:10.1303/jjaez.41.75)

18. Noda, H. 1986 Pre-mating flight of rice planthopper migrants (Homoptera: Delphacidae) collected on the East China Sea. *Appl. Entomol. Zool.* **21**, 175–176. (doi:10.1303/aez.21.175)

19. Katayama, E. 1975 The relation between ovarial development and copulation in a leafhopper and planthoppers of rice plant (Homoptera: Auchenorrhyncha). *Jpn. J. Appl. Entomol. Zool.* **19**, 176–181.

20. Willard, J. R. 1977 Dispersal of California red scale (*Aonidiella aurantii* (Maskell))(Homoptera: Diaspididae) in relation to weather variables. *Aust. J. Entomol.* **15**, 395–404.

21. Kobori, Y., Nakata, T. & Ohto, Y. 2011 Estimation of dispersal pattern of adult Asian citrus psyllid, *Diaphorina citri* Kuwayama (Hemiptera: Psyllidae). *Jpn. J. Appl. Entomol. Zool.* **55**, 177–181. (doi:10.1303/jjaez.2011.177)

22. Johnson, C. G. 1969 Migration and dispersal of insects by flight. *Migr. Dispersal Insects Flight*

23. Ito, K. 1982 Immigration of *Cletus punctiger* Dallas (Heteroptera: Coreidae) into paddy field in relation to food plant preference in the adult. *Jpn. J. Appl. Entomol. Zool.* **26**, 300–304. (doi:10.1303/jjaez.26.300)

24. Takahashi, A. & Higuchi, H. 2006 Copulation frequency and reproduction of the rice leaf bug, *Trigonotylus caelestialium* (Kirkaldy) (Heteroptera: Miridae). *Jpn. J. Appl. Entomol. Zool.* **50**, 225–229. (doi:10.1303/jjaez.2006.225)

25. Okutani-Akamatsu, Y., Watanabe, T. & Azuma, M. 2009 Mating behavior and oviposition of the sorghum plant bug, *Stenotus rubrovittatus* (Matsumura), (Heteroptera: Miridae) under laboratory conditions. *Jpn. J. Appl. Entomol. Zool.* **53**, 13–20. (doi:10.1303/jjaez.2009.13)

26. Ware, A. B. & Compton, S. G. 1994 Dispersal of adult female fig wasps: 2. Movements between trees. *Entomol. Exp. Appl.* **73**, 231–238. (doi:10.1111/j.1570-7458.1994.tb01860.x)

27. Östrand, F. & Anderbrant, O. 2001 Mating duration and frequency in a pine sawfly. *J. Insect Behav.* **14**, 595–606.

28. King, B. 1993 Flight activity in the parasitoid wasp *Nasonia vitripennis* (Hymenoptera: Pteromalidae). *J. Insect Behav.* **6**, 313–321.

29. Whiting, A. R. 1967 The biology of the parasitic wasp *Mormoniella vitripennis* [= *Nasonia brevicornis*] (Walker). *Q. Rev. Biol.* , 333–406.

30. Qureshi, J. A., Buschman, L. L., Throne, J. E. & Ramaswamy, S. B. 2006 Dispersal of adult *Diatraea grandiosella* (Lepidoptera: Crambidae) and its implications for corn borer resistance management in *Bacillus thuringiensis* maize. *Ann. Entomol. Soc. Am.* **99**, 279–291.

31. Showers, W. B., Hellmich, R. L., Derrick-Robinson, M. E. & Hendrix, W. H. 2001 Aggregation and dispersal behavior of marked and released European corn borer (Lepidoptera: Crambidae) adults. *Environ. Entomol.* **30**, 700–710.

32. Hunt, T. E., Higley, L. G., Witkowski, J. F., Young, L. J. & Hellmich, R. L. 2001 Dispersal of adult European corn borer (Lepidoptera: Crambidae) within and proximal to irrigated and non-irrigated corn. *J. Econ. Entomol.* **94**, 1369–1377.

33. Dalecky, A., Ponsard, S., Bailey, R. I., Pélissier, C. & Bourguet, D. 2006 Resistance evolution to Bt crops: predispersal mating of European corn borers. *PLoS Biol* **4**, e181.

34. Cuming, F. G. 1961 The distribution, life history, and economic importance of the winter moth, *Operophtera brumata* (L.)(Lepidoptera, Geometridae) in Nova Scotia. *Can. Entomol.* **93**, 135–142.

35. Edland, T. & others 1971 Wind dispersal of the winter moth larvae *Operophtera brumata* L.(Lep., Geometridae) and its relevance to control measures. *Entomol Tidsskr* **18**, 103–107.

36. Shapiro, I. D. 1977 Interaction of population biology and mating behavior of the fiery skipper, *Hylephila phylaeus* (Hesperiidae). *Am. Midl. Nat.* , 85–94.

37. Cameron, P. J., Wallace, A. R., Madhusudhan, V. V., Wigley, P. J., Qureshi, M. S. & Walker, G. P. 2005 Mating frequency in dispersing potato tuber moth, *Phthorimaea operculella*, and its influence on the design of refugia to manage resistance in Bt transgenic crops. *Entomol. Exp. Appl.* **115**, 323–332.

38. Wakamura, S., Kozai, S., Kegasawa, K. & Inoue, H. 1990 Population dynamics of adult *Spodoptera litura* (Fabricius)(Lepidoptera: Noctuidae): dispersal distance of male moths and its seasonal change. *Appl. Entomol. Zool.* **25**, 447–456.

39. Tu, Y.-G., Wu, K.-M., Xue, F.-S. & Lu, Y.-H. 2010 Laboratory evaluation of flight activity of the common cutworm, *Spodoptera litura* (Lepidoptera: Noctuidae). *Insect Sci.* **17**, 53–59.

40. Ohsaki, N. 1980 Comparative population studies of threePieris butterflies, *P. rapae*, *P. melete* and *P. napi*, living in the same area. *Res. Popul. Ecol.* **22**, 163–183.

41. Shapiro, A. M. 1970 The role of sexual behavior in density-related dispersal of pierid butterflies. *Am. Nat.* , 367–372.

42. Inoue, H., Kamiwada, H. & Fukamachi, S. 2004 Seasonal changes in adult density and female mating status of the rice leaf roller *Cnaphalocrocis medinalis* Guenée (Lepidoptera: Pyralidae) in paddy fields of Southern Kyushu, Japan. *Jpn. J. Appl. Entomol. Zool. Jpn.*

43. Shirai, Y. & Nakamura, A. 1994 Dispersal movement of male adults of the diamondback moth, *Plutella xylostella* (Lepidoptera: Yponomeutidae), on cruciferous vegetable fields, studied using the mark-recapture method. *Appl. Entomol. Zool.* **29**, 339–348.

44. Green, G. W. & Pointing, P. J. 1962 Flight and dispersal of the European pine shoot moth, *Rhyacionia buoliana* (Schiff.) II. Natural dispersal of egg-laden females. *Can. Entomol.* **94**, 299–314.

45. Diss, A. L., Kunkel, J. G., Montgomery, M. E. & Leonard, D. E. 1996 Effects of maternal nutrition and egg provisioning on parameters of larval hatch, survival and dispersal in the gypsy moth, *Lymantria dispar* L. *Oecologia* **106**, 470–477.

46. Schumacher, P., Weyeneth, A., Weber, D. C. & Dorn, S. 1997 Long flights in *Cydia pomonella* L. (Lepidoptera: Tortricidae) measured by a flight mill: influence of sex, mated status and age. *Physiol. Entomol.* **22**, 149–160.

47. Popov, G. B. 1954 Notes on the behaviour of swarms of the desert locust (*Schistocerca gregaria* Forskål) during oviposition in Iran. *Trans. R. Entomol. Soc. Lond.* **105**, 65–77.

48. Popov, G. B. 1958 Ecological studies on oviposition by swarms of the desert locust (*Schistocerca gregaria* Forskal) in eastern Africa. *Anti-Locust Bull*

49. Gill, H. K., Garg, H., Gill, A. K., Gillett-Kaufman, J. L. & Nault, B. A. 2015 Onion thrips (Thysanoptera: Thripidae) biology, ecology, and management in onion production systems. *J. Integr. Pest Manag.* **6**, 6.

50. Mound, L. A. 2005 Thysanoptera: diversity and interactions. *Annu Rev Entomol* **50**, 247–269.

51. Osakabe, M., Isobe, H., Kasai, A., Masuda, R., Kubota, S. & Umeda, M. 2008 Aerodynamic advantages of upside down take-off for aerial dispersal in Tetranychus spider mites. *Exp. Appl. Acarol.* **44**, 165–183. (doi:10.1007/s10493-008-9141-2)

52. Croft, B. A. & Jung, C. 2001 Phytoseiid dispersal at plant to regional levels: a review with emphasis on management of *Neoseiulus fallacis* in diverse agroecosystems. *Exp. Appl. Acarol.* **25**, 763–784.
