# Supplementary material for: Optimal management strategy of insecticide resistance under various insect life histories: Heterogeneous timing of selection and interpatch dispersal
Source: Evol Appl. 2017 Nov 2;11(2):271–83. doi: 10.1111/eva.12550 (PMC5775500; doi:10.1111/eva.12550)
Supplement: Supplementary file 11 [file EVA-11-271-s011.docx]

## Online Appendix A: Oviposition model

The egg genotype frequencies are calculated based on the random mating assumption by converting the female densities into gamete frequencies, recombining the male and female gametes into egg genotype frequencies, and then calculating back to densities. We define $\mathbf{P}_{i,j}^{\text{female}}\left(\tau\right)$ as the vector of gamete frequencies of females which mated in patch $i$ and present at patch $j$,

$$\mathbf{P}_{i,j}^{\text{female}}\left(\tau\right)=\frac{1}{\sum_{x\in G} n_{x,i,j}^{\text{VII}}\left(\tau\right)}\left(\begin{matrix} n_{S^{\text{A}}S^{\text{A}}S^{\text{B}}S^{\text{B}},i,j}^{\text{VII}}\left(\tau\right)+\frac{1}{2}\left(n_{S^{\text{A}}R^{\text{A}}S^{\text{B}}S^{\text{B}},i,j}^{\text{VII}}\left(\tau\right)+n_{S^{\text{A}}S^{\text{A}}S^{\text{B}}R^{\text{B}},i,j}^{\text{VII}}\left(\tau\right)\right)+\frac{1}{4}n_{S^{\text{A}}R^{\text{A}}S^{\text{B}}R^{\text{B}},i,j}^{\text{VII}}\left(\tau\right) \\ n_{R^{\text{A}}R^{\text{A}}S^{\text{B}}S^{\text{B}},i,j}^{\text{VII}}\left(\tau\right)+\frac{1}{2}\left(n_{S^{\text{A}}R^{\text{A}}S^{\text{B}}S^{\text{B}},i,j}^{\text{VII}}\left(\tau\right)+n_{R^{\text{A}}R^{\text{A}}S^{\text{B}}R^{\text{B}},i,j}^{\text{VII}}\left(\tau\right)\right)+\frac{1}{4}n_{S^{\text{A}}R^{\text{A}}S^{\text{B}}R^{\text{B}},i,j}^{\text{VII}}\left(\tau\right) \\ n_{S^{\text{A}}S^{\text{A}}R^{\text{B}}R^{\text{B}},i,j}^{\text{VII}}\left(\tau\right)+\frac{1}{2}\left(n_{S^{\text{A}}R^{\text{A}}R^{\text{B}}R^{\text{B}},i,j}^{\text{VII}}\left(\tau\right)+n_{S^{\text{A}}S^{\text{A}}S^{\text{B}}R^{\text{B}},i,j}^{\text{VII}}\left(\tau\right)\right)+\frac{1}{4}n_{S^{\text{A}}R^{\text{A}}S^{\text{B}}R^{\text{B}},i,j}^{\text{VII}}\left(\tau\right) \\ n_{R^{\text{A}}R^{\text{A}}R^{\text{B}}R^{\text{B}},i,j}^{\text{VII}}\left(\tau\right)+\frac{1}{2}\left(n_{S^{\text{A}}R^{\text{A}}R^{\text{B}}R^{\text{B}},i,j}^{\text{VII}}\left(\tau\right)+n_{R^{\text{A}}R^{\text{A}}S^{\text{B}}R^{\text{B}},i,j}^{\text{VII}}\left(\tau\right)\right)+\frac{1}{4}n_{S^{\text{A}}R^{\text{A}}S^{\text{B}}R^{\text{B}},i,j}^{\text{VII}}\left(\tau\right) \end{matrix}\right).$$

Likewise, $\mathbf{P}_{i}^{\text{male}}\left(\tau\right)$, the male gamete frequencies (stored as sperm in female body) are calculated based on the patch of mating. The egg genotype frequencies for each of the female types, $\mathbf{B}_{i,j}$, are calculated as products among elements of those vectors representing gamete frequencies from females, $\mathbf{P}_{i,j}^{\text{female}}(\tau)$ and from males, $\mathbf{P}_{i}^{\text{male}}(\tau)$, as follows,

$$\mathbf{B}_{i,j}=\left(\begin{matrix} \mathbf{P}_{i,j}^{\text{female}}\left[1\right]\mathbf{P}_{i}^{\text{male}}\left[1\right] \\ \mathbf{P}_{i,j}^{\text{female}}\left[1\right]\mathbf{P}_{i}^{\text{male}}\left[2\right]+\mathbf{P}_{i,j}^{\text{female}}\left[2\right]\mathbf{P}_{i}^{\text{male}}\left[1\right] \\ \mathbf{P}_{i,j}^{female}\left[2\right]\mathbf{P}_{i}^{male}\left[2\right] \\ \mathbf{P}_{i,j}^{female}\left[1\right]\mathbf{P}_{i}^{male}\left[3\right]+\mathbf{P}_{i,j}^{female}\left[3\right]\mathbf{P}_{i}^{male}\left[1\right] \\ \mathbf{P}_{i,j}^{\text{female}}\left[1\right]\mathbf{P}_{i}^{\text{male}}\left[4\right]+\mathbf{P}_{i,j}^{\text{female}}\left[2\right]\mathbf{P}_{i}^{\text{male}}\left[3\right]+\mathbf{P}_{i,j}^{\text{female}}\left[3\right]\mathbf{P}_{i}^{\text{male}}\left[2\right]+\mathbf{P}_{i,j}^{\text{female}}\left[4\right]\mathbf{P}_{i}^{\text{male}}\left[1\right] \\ \mathbf{P}_{i,j}^{\text{female}}\left[2\right]\mathbf{P}_{i}^{\text{male}}\left[4\right]+\mathbf{P}_{i,j}^{\text{female}}\left[4\right]\mathbf{P}_{i}^{\text{male}}\left[2\right] \\ \mathbf{P}_{i,j}^{\text{female}}\left[3\right]\mathbf{P}_{i}^{\text{male}}\left[3\right] \\ \mathbf{P}_{i,j}^{\text{female}}\left[3\right]\mathbf{P}_{i}^{\text{male}}\left[4\right]+\mathbf{P}_{i,j}^{\text{female}}\left[4\right]\mathbf{P}_{i}^{\text{male}}\left[3\right] \\ \mathbf{P}_{i,j}^{\text{female}}\left[4\right]\mathbf{P}_{i}^{\text{male}}\left[4\right] \end{matrix}\right).$$

Finally, we can recover $n_{x,i}^{\text{egg}}\left(\tau+1\right)$, the densities for the egg genotypes in each patch $i$, as the corresponding row of $r\left(\sum_{x\in G} n_{x,i,\text{T}}^{\text{VII}}\left(\tau\right)\mathbf{B}_{i,\text{T}}+\sum_{x\in G} n_{x,i,\text{N}}^{\text{VII}}\left(\tau\right)\mathbf{B}_{i,\text{N}}\right)$. The first and second terms indicate contributions of the females that mated in the treated patch and the refuge, respectively, to eggs on the patch $i$ at the next generation.

## Online Appendix B: Effect of population reproduction rate on resistance evolution

Low fecundity $r$ in the pest insect leads to a slow recovery of the population after an insecticide(s) application in the treated field. If the local population size is small enough, the effects of density-dependence are negligible both in the treated and refuge patches, which makes the relative size of the treated to refuge population independent of $r$, and the waiting time until resistance is scarcely affected by $r$.

As $r$ increases, density-dependence cancels the difference in the population densities between the two areas, even after the pesticide application; the refuge is no longer able to supply the susceptible individuals in some life-history types (e.g. in the Supplementary Figure S9: the case of high efficacy in juvenile selection $s_{\text{j}}=0$ and with the post-mating dispersal). On the other hand, mixture strategy performs well even with the large $r$ at certain conditions with incomplete selection ($s>0$).

When $r$ is low, the pest insect population in the treated field can become extinct in some parameter regions (Supplementary Figure S9). As $r$ determines the recovery rate of the population lost in the selection, such a local extinction occurs when pesticide resistance evolves slowly. Therefore, the mixture strategy often leads to population extinction at higher $h$ values than the other two strategies, because of the effective removal of insects from the treated-field population.

## Captions for the online supplementary materials

Supplementary Table S1: In another supplementary file.

Supplementary Figure S1. Dominance of *R* alleles, insect life-histories and insecticide-application efficacy affecting the waiting-time to resistance ($R^{\text{B}}$ allele-frequency reaches 50% in the treated field), provided insecticides are used in Sequential (black thin line), Mixture (red broken) and Rotation (blue dotted) strategies. This figure is the counterpart of Figure 2 (main text) with the difference that the refuge-area proportion is 0.1 here while 0.5 in Figure 2. Other parameters are same as Figure 2. In the plots of row 8, endpoints with open circles signify the local extinction of the population (population density in the treated patch reached below the limit of calculation, $10^{-20}(1-k)K$, before resistance evolved).

Supplementary Figure S2. *R*-allele frequencies in the treated patch just before mating of the 4th generation adults. The two insecticides are used in the sequential (pesticide A: black thin line), mixture (pesticide A: red broken), and rotation strategies (pesticide A: blue dotted, B: blue dashed: in the rotation strategy, pesticide B is used at 4th generation).

Supplementary Figure S3. Optimal management strategies under variation in dominance of the resistance alleles and proportional area of the refuge patch, assuming no fitness-cost and no cross-resistance. Success of a management strategy was defined as the waiting-time until $R^{\text{B}}$ allele-frequency reaches 50% in the treated field, and then compared between the three strategies; the difference less than 2.0 times was assigned to “No difference.” Broken lines in each plot show the default values of dominance of *R* genes (0.1) and the proportional area (0.5) in this study, respectively.

Supplementary Figure S4. Optimal management strategies under variation in selection efficacy. Dominance and refuge-area proportion are fixed at 0.1 and 0.5, respectively. Assuming no fitness-costs and cross resistance between the resistance genes. Results are classified in the same manner as in Fig. S3.

Supplementary Figure S5. Optimal management strategies under variation in dispersal rates. Dominance and refuge-area proportion are fixed at 0.1 and 0.5, respectively. Assuming no fitness-costs and cross-resistance between the resistance genes. Results are classified in the same manner as in Fig. S3.

Supplementary Figure S6. Optimal management strategy under variation in dominance of resistance genes and the degree of cross-resistance. Cross-resistance was assumed to be reciprocal: i.e. an individual having $R^{\text{A}}$ allele(s) survive under exposure to pesticide B at a certain probability (= the degree of cross resistance), and vice versa. Results are shown in the same manner as in Fig. S3, although the refuge area proportion is fixed at 0.5.

Supplementary Figure S7. Decreased relative fitness of the resistant homozygotes, $R^{\text{B}}R^{\text{B}}$, affecting the waiting-time to resistance. The hatching probability of an $R^{\text{B}}R^{\text{B}}$ egg varied from 1.0 to 0.0 while all eggs hatched in $S^{\text{B}}S^{\text{B}}$ homozygotes and $S^{\text{B}}R^{\text{B}}$ heterozygotes (hatching probability of 1.0). No fitness-cost on pesticide A. Dominance and refuge-area proportion are fixed at 0.1 and 0.5, respectively. All other parameters are set as default values (Table 1). Some values are not plotted for large fitness cost because of local extinction (population density reached below $10^{-20}(1-k)K$ before resistance evolved).

Supplementary Figure S8. Optimal management strategies considering the fitness cost on pesticide B, which is defined as a decline in the egg-hatching success of the resistant homozygotes, $R^{\text{B}}R^{\text{B}}$, to 90% ($\zeta_{\text{resB}}=0.9$). No fitness-cost on pesticide A. Results are shown in the same manner as in Fig. S3.

Supplementary Figure S9. The effects of $r$ (fecundity) on the generation time until resistance evolved ($R^{\text{B}}$-allele frequency of 50% in the treated area). Some values are not plotted for small $r$ because of local extinction (population density reached below $10^{-20}(1-k)K$ before resistance evolved). The dominance of the resistance genes, $h$, and refuge proportion, $k$, were fixed at 0.1 and 0.5, respectively.
